# Review on Graphene-, Graphene Oxide-, Reduced Graphene Oxide-Based Flexible Composites: From Fabrication to Applications

**DOI:** 10.3390/ma15031012

**Published:** 2022-01-28

**Authors:** Aamir Razaq, Faiza Bibi, Xiaoxiao Zheng, Raffaello Papadakis, Syed Hassan Mujtaba Jafri, Hu Li

**Affiliations:** 1 Department of Physics, COMSATS University Islamabad, Lahore Campus 54000, Pakistan; faiza4563@gmail.com; 2 Shandong Technology Centre of Nanodevices and Integration, School of Microelectronics, Shandong University, Jinan 250101, China; 202120353@mail.sdu.edu.cn; 3 TdB Labs AB, Uppsala Business Park, 75450 Uppsala, Sweden; rafpapadakis@gmail.com; 4 Department of Chemistry, Uppsala University, 75120 Uppsala, Sweden; 5 Department of Electrical Engineering, Mirpur University of Science and Technology (MUST), Mirpur Azad Jammu and Kashmir 10250, Pakistan; hassan.jafri@must.edu.pk; 6 Ångström Laboratory, Department of Materials Science and Engineering, Uppsala University, 75121 Uppsala, Sweden

**Keywords:** graphene, flexible devices, composite, graphene oxide, reduced graphene oxide

## Abstract

In the new era of modern flexible and bendable technology, graphene-based materials have attracted great attention. The excellent electrical, mechanical, and optical properties of graphene as well as the ease of functionalization of its derivates have enabled graphene to become an attractive candidate for the construction of flexible devices. This paper provides a comprehensive review about the most recent progress in the synthesis and applications of graphene-based composites. Composite materials based on graphene, graphene oxide (GO), and reduced graphene oxide (rGO), as well as conducting polymers, metal matrices, carbon–carbon matrices, and natural fibers have potential application in energy-harvesting systems, clean-energy storage devices, and wearable and portable electronics owing to their superior mechanical strength, conductivity, and extraordinary thermal stability. Additionally, the difficulties and challenges in the current development of graphene are summarized and indicated. This review provides a comprehensive and useful database for further innovation of graphene-based composite materials.

## 1. Introduction

It is well known that materials play an important role in the development of science and technology, because the realization of a new technology often requires the support of novel materials. Therefore, exploring materials with excellent properties has always been an important subject of scientific research. A remarkable material, graphene, has attracted widespread attention since it was first exfoliated from graphite by Andre Geim and Konstantin Novoselov in 2004. As a result of its prominent performances, graphene can be used in various fields, such as energy storage, biosensing, optoelectronics, flexible electronics, electrochemical sensing, robotics, textile industry, and so on [1,2,3,4]. The discovery of graphene marked the beginning of a new era in material science research [5]. 

**Figure 1:**
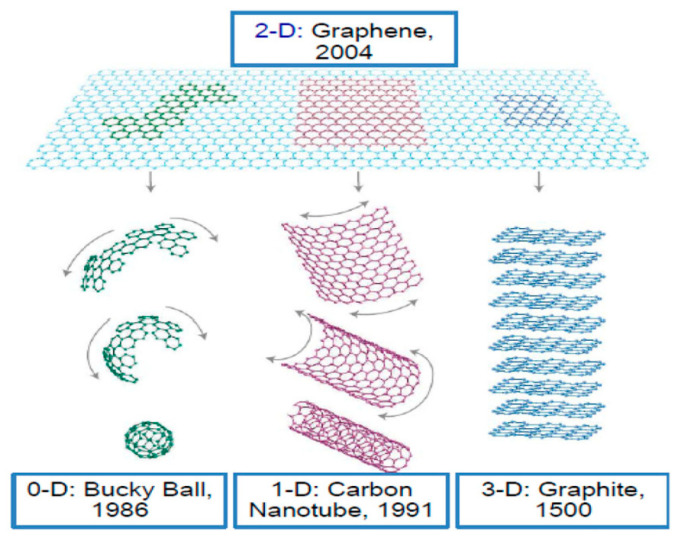
Various structures of graphene (0D bucky ball, 1D carbon nanotube, 3D graphite). (Reproduced with permission from ref. [9]. Copyright 2016 Springer Publications).

**Figure 2:**
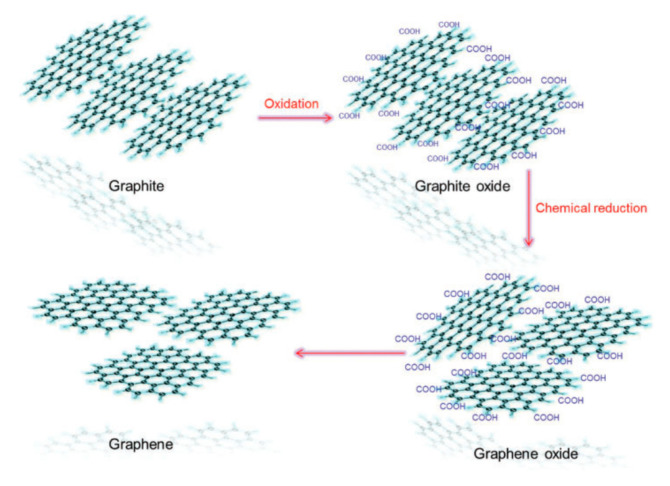
Schematic of the transformation of graphite oxide to GO and graphene. (Reproduced with permission from ref. [6]. Copyright 2016 SAGE Publications).

Graphene with a thickness of a single carbon atom is arranged in a honeycomb lattice. It is very solid and can be fashioned into 0D, 1D, 3D forms (Figure 1) [6]. In addition, it is extraordinary transparent and possesses high crystallite as well as outstanding electronic properties. Although graphene has many excellent properties, there is no bandgap in graphene, and it has poor water solubility, which greatly limits its application in some areas [7]. An effective way to overcome these limitations and expand the range of application of graphene is to prepare graphene derivatives. For example, treating graphite with strong oxidants will add epoxy groups, hydroxyl groups, and carboxyl group on the basal plan of graphite layers, thus producing graphene oxide (GO). These polar oxygen-containing functional groups make GO highly hydrophilic. This allows GO to have excellent dispersibility in many solvents, especially in water. In addition, the oxygen-containing functional groups can provide reactive sites for chemical modification or functionalization of GO, which in turn can be used to develop GO-based materials. Although the oxygen-containing groups can obviate some disadvantages of graphene, they also cause some problems. For example, they make GO electrically insulating. Nevertheless, the chemical reduction of GO can restore its conductivity to some extent. The obtained reduced GO (rGO) still carries some functional groups, which results in a good dispersion of rGO in many solvents. Most importantly, it is relatively easy to control the electrical performance and solubility of rGO by controlling the number of the remaining functional groups. The properties of this chemically reduced graphene approximately resemble those of pristine graphene [6,8]. The transformation of graphite to graphite oxide, GO and graphene is shown in Figure 2.

Graphene and its derivatives have their own unique advantages and can be used in many domains applying different techniques, such as thermal chemical vapor deposition (CVD), self-assembly technique, spin coating, vacuum filtration, thermal decomposition, solution dispersion technique, and chemical decomposition polymer processing technique [8,10,11,12,13,14,15]. This review comprises of synthesis of composite material based on graphene and its derivatives along with chemical properties, and mainly focus on the potential applications of these materials.

## 2. Synthesis of Graphene and Its Derivatives

### 2.1. GO

**Figure 3:**
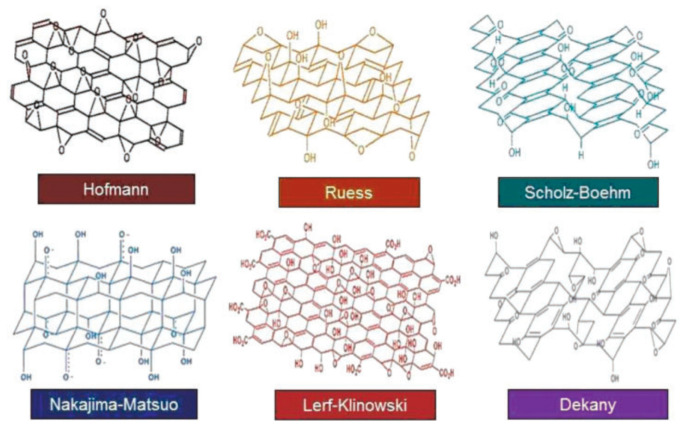
Different structures of GO. (Reproduced with permission from ref. [6]. Copyright 2016 SAGE Publications).

**Figure 4:**
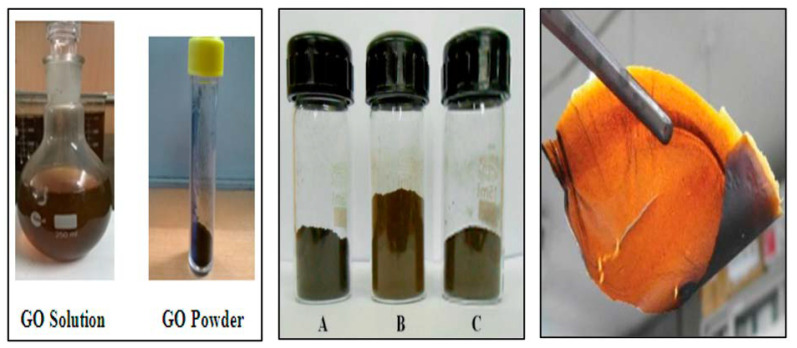
GO suspension (left side), powder, and flexible sheet (right side). (Reproduced with permission from ref. [18,21]. Copyright 2015 JNMNT and 2014 Hindawi Publications).

There are many reports about the synthesis of GO, and the structures of the obtained products are slightly different (Figure 3). One of the most classical methods was proposed by Williams Hummers JR and Richard Offeman in 1958. The general process was as follows. Graphite was first mixed with concentrated sulfuric acid and oxidizers such as sodium nitrate, then potassium permanganate was added under a precise temperature control, followed by the addition of reducing and reaction stopping agents such as hydrogen peroxide at the end of the process [16]. This method supplies a high yield of colloidal suspension and powdery product [17]. Later, numerous research groups made further improvements of the preparation method focusing on three main parameters, i.e., precursors ratio, time, and temperature [18]. For example, Marcano et al. synthesized GO by using the Tours method and obtained high-quality GO by adding phosphoric acid as a key precursor and removing sodium nitrate with the product. This method is better than previous methods due to its simplicity and outstanding product quality [19]. In addition to these three parameters, the size of graphite particle also has a great effect on the quality of the final products [20]. According to the demands of different applications, various physical forms of GO such as suspension, powder, and flexible sheet can be prepared. The corresponding photos are shown in Figure 4. 

### 2.2. Graphene 

Several attempts have been adopted to study the synthesis methods of carbon-based materials. The very first attempt dates back to 1962. Boehm et al. prepared soot composed of thin-layer graphite-intercalated compounds by the reduction and combustion of graphite oxide. In 1944, these products were named as graphene platelets and had a single carbon layer [22]. In 2004, Novoselov et al. obtained graphene by the scotch tape method and won the Noble Prize in 2010 [23]. Till now, the preparation methods of graphene include top-down and bottom-up techniques. Top-down methods include scotch tape exfoliation, liquid-phase exfoliation, and chemical synthesis. Bottom-up methods mainly comprise CVD and molecular beam epitaxy [24,25,26]. In this section, typical methods of graphene synthesis will be introduced.

#### 2.2.1. Exfoliation and Cleavage

**Figure 5:**
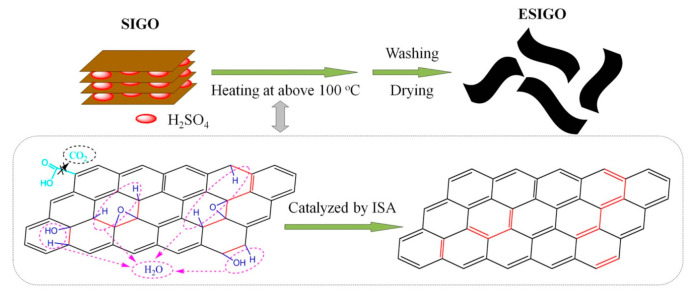
Schematic of the H_2_SO_4_-intercalated GO (SIGO) process. (Reproduced with permission from ref. [28]. Copyright 2013 Springer Nature Publications).

Micromechanical cleavage is a process in which the bonds in graphite crystal are broken by mechanical energy so that graphene sheets are peeled from a silicon substrate. Exfoliation can be done in solution by intercalating graphite and exfoliating to a single carbon sheet [27]. GO prepared by the conventional Hummer’s method acts as a precursor for the preparation of graphene sheets when intercalated with sulphuric acid. The process involves the reduction and expansion of sulphuric acid-intercalated graphite oxide for the large-scale production of graphene. As shown in Figure 5, when the slurry obtained by Hummer’s method is placed in a box furnace, graphite oxide can be expanded into graphene [28].

#### 2.2.2. Thermal CVD Techniques

**Figure 6:**
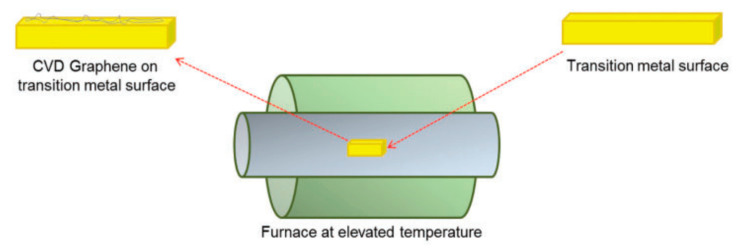
Graphene synthesis via the CVD method. (Reproduced with permission from ref. [6]. Copyright 2016 SAGE Publications).

The CVD technique is another common method used to synthesize graphene. Certain carrier gases and carbon-based precursors like camphor and methane are injected into a CVD chamber at a specific temperature. Then, the carbon precursor is decomposed to form graphene on transition metal sheets such as a nickel foam (Figure 6). In addition to exfoliation and the CVD method, there are many other methods that can be used to prepare graphene, such as thermal decomposition of SiC [10] and others.

### 2.3. rGO

**Figure 7:**
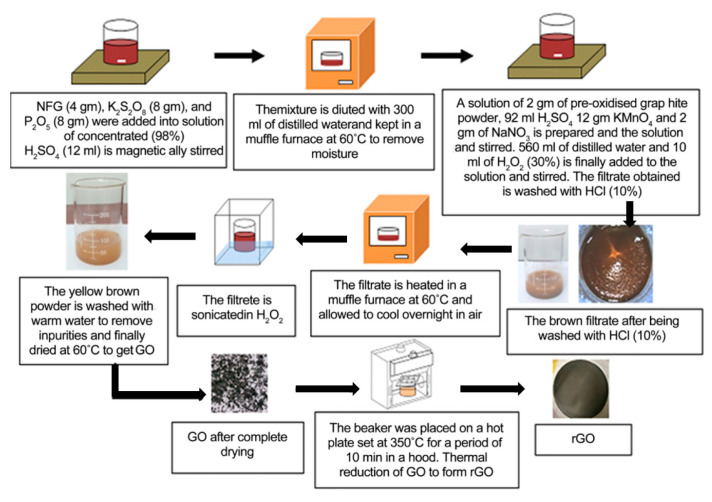
Steps of the synthesis of GO and rGO. (Reproduced with permission from ref. [29]. Copyright 2017 Scientific Research Publications).

**Figure 8:**
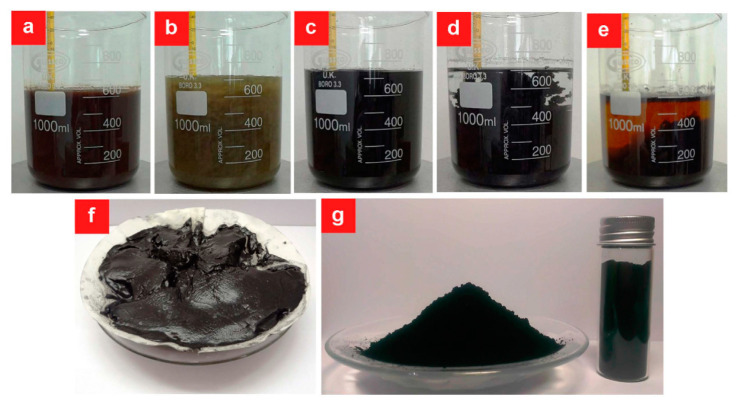
Consecutive steps in the chemical synthesis of rGO using ascorbic acid as a reducing agent. (**a**) Oxidation and exfoliation of graphite using Hummer’s method. (**b**) Reduction and conversion of Mn (VII) ions to soluble Mn (II) ions by the addition of ascorbic acid. (**c**) Color transition of the exfoliated graphite oxide from greenish yellow to black in the early stage of reduction. (**d**) Loss of hydrophilicity of GO when stirring is paused. (**e**) Precipitation of rGO after completion of the reduction stage and cooling down to room temperature. (**f**) Filtration of rGO using cellulose filter paper. (**g**) rGO powder after freeze-drying. (Reproduced with permission from ref. [31]. Copyright 2015 Springer Nature Publications).

GO prepared by Hummer’s method consists of a-few-layer carbon platelets decorated with oxygen containing functional groups. The removal of some oxygen-based groups by reducing agents or thermal treatment can yield rGO (Figure 7). The main process is as follows. GO is exfoliated via ultrasonication and then reduced by hydrazine hydrate, a strong reducing agent, for 2 h. Since hydrazine is toxic, alternative reagents such as NaBH_4_, ascorbic acid, and HI can be used. Among these, ascorbic acid is essential for the scalable production of rGO. The chemical procedure to obtain rGO using ascorbic acid as a reducing agent is shown in Figure 8. This reaction does not produce toxic gases [29]. rGO has been proven to be a good candidate for various applications such as field effect transistors (FET), solar cells, energy applications, and production of composite paper-like materials [30] due to its abundant atomic defects.

## 3. Flexible Graphene Composites: From Fabrication to Applications

With the evolution of science and technology, more and more novel materials with fascinating properties have been discovered and can be applied in many domains. Among these novel materials, graphene has received a lot of attention because of its excellent properties, such as high mechanical strength, stability, charge storage capacity, etc. Furthermore, graphene has very good flexibility and shows excellent application prospects in some flexible composite materials. For instance, a flexible composite consisting of polyethylene–ioxythiophene–graphene was fabricated by the following method. First, PtCl_4_ was added to an NaOH solution under stirring followed by heating at 160 °C for 3 h. Next, the solution was treated with 2 M sulphuric acid and ethyl glycol and then was electrochemically deposited on a graphene-filtrated carbon cloth/graphene paper substrate. It is worth noting that this flexible composite material is expected to be used in energy storage, because the square shape of the corresponding electrochemical graph indicates excellent capacitive properties [32]. Likewise, when polyaniline (PANI), a conducting polymer with good stability, was mixed with graphene in the form of nanofibers by the vacuum filtration method, the obtained composite film showed not only excellent flexibility but also good electrochemical stability [33]. 

Many additional related reports on the preparation and application of other flexible graphene-based composites have been published [34]. For example, by regulating the ratio of each components, poly(3,4-ethylenedioxythiophene)/poly(4-styrenesulfonate) (PEDOT/PSS)/graphene composites can be fabricated; they show great potential applications in energy-harvesting systems such as thermoelectric devices and solar cells [35]. Besides, a flexible composite was prepared by simple coating MnO_2_ on Zn_2_SnO_4_ (ZTO) nanowires grown on carbon microfibers. This material can be used in supercapacitor electrodes, whose composite analysis suggests a long cycle life [36]. A typical rectangular voltammogram can be seen for carbon cloth and graphene-coated carbon cloth with electrodeposited PEDOT. This result indicates that graphene-based materials have excellent electrical performance and can be excellent electrode materials in energy storage devices. A simple spin coating technique used at ambient conditions for the fabrication of graphene-based transparent electrodes was proposed. In this method, a graphene slurry was added to dimethyl sulfoxide (DMSO) and then to a pure PEDOT/PSS aqueous solution. Then, a spin coater was used to spin the coating, and the product was left to rest at room temperature [37]. Graphene/MnO_2_ combined with light-weight carbon nanotubes (CNTs) formed an ultra-flexible thin-film composite, which has been used for various energy storage devices as a robust electrode, as it holds extraordinary mechanical properties with superb electrochemical activities when fabricated by the chemical co-precipitation method [38]. 

**Figure 9:**
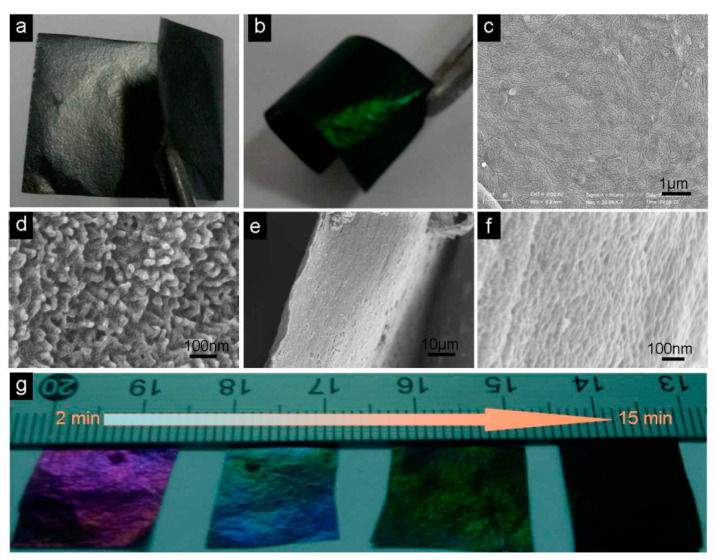
(**a**) Flexible graphene paper with the size of 8 × 5 cm. (**b**) Graphene/PANI paper (3 cm × 1.5 cm), electrochemical deposition time of 10 min. (**c**,**d**) SEM images of the surface of graphene/PANI paper at different magnifications. (**e**,**f**) SEM images of cross sections of graphene/PANI paper at different magnifications. (**g**) Graphene/PANI composite papers with different electropolymerization times (From left to right: 2, 5, 10, 15 min). (Reproduced with permission from ref. [39]. Copyright 2013 RSC Publications).

**Figure 10:**
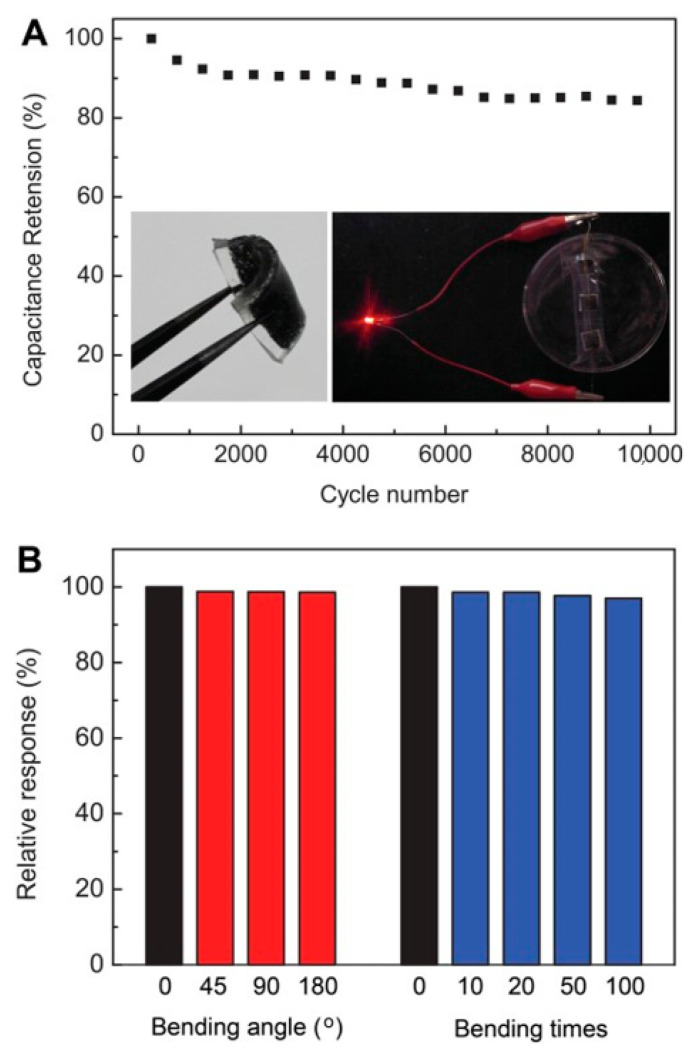
(**A**) Cyclic behavior of MnO_2_/ERGO//CNT ERGO. (**B**) Specific capacitance retention ratio of the flexible supercapacitor after inward bending by different angles or repeated bending. (Reproduced with permission from ref. [41]. Copyright 2014 Wiley Publications).

Beside flexibility, the light weight and the efficiency of a device are also very important. To meet the current energy demand and increase the performance of energy devices, paper-based electrodes of graphene/PANI composite have been reported. They were prepared by the electropolymerization of PANI on graphene paper [39]. As shown in Figure 9a,b, graphene/PANI paper retains the origin flexibility of graphene paper. Graphene/PANI paper as a supercapacitor electrode exbibits a high specific capacitance and excellent cycling stability due to the uniform growth of PANI on graphene (Figure 9c–f); it has great potential for application in the construction of portable energy devices. Light-weight and flexible graphene/polypyrrole (PPy) fibers were fabricated by spinning GO and pyrrole in a FeCl_3_ solution, which helped to control the diameter of fiber, finally obtaining graphene/PPy fibers [40]. MnO_2_ can also be used for the fabrication of this composite due to its high specific capacitance. A 3D graphene/MnO_2_ composite foam to be used as a negative electrode for asymmetric supercapacitors was fabricated by the solution casting method. GO was reduced on Ni foam and then subjected to electrodeposition of MnO_2_ to obtain an asymmetric supercapacitor, showing excellent cyclic stability (Figure 10) [41].

**Figure 11:**
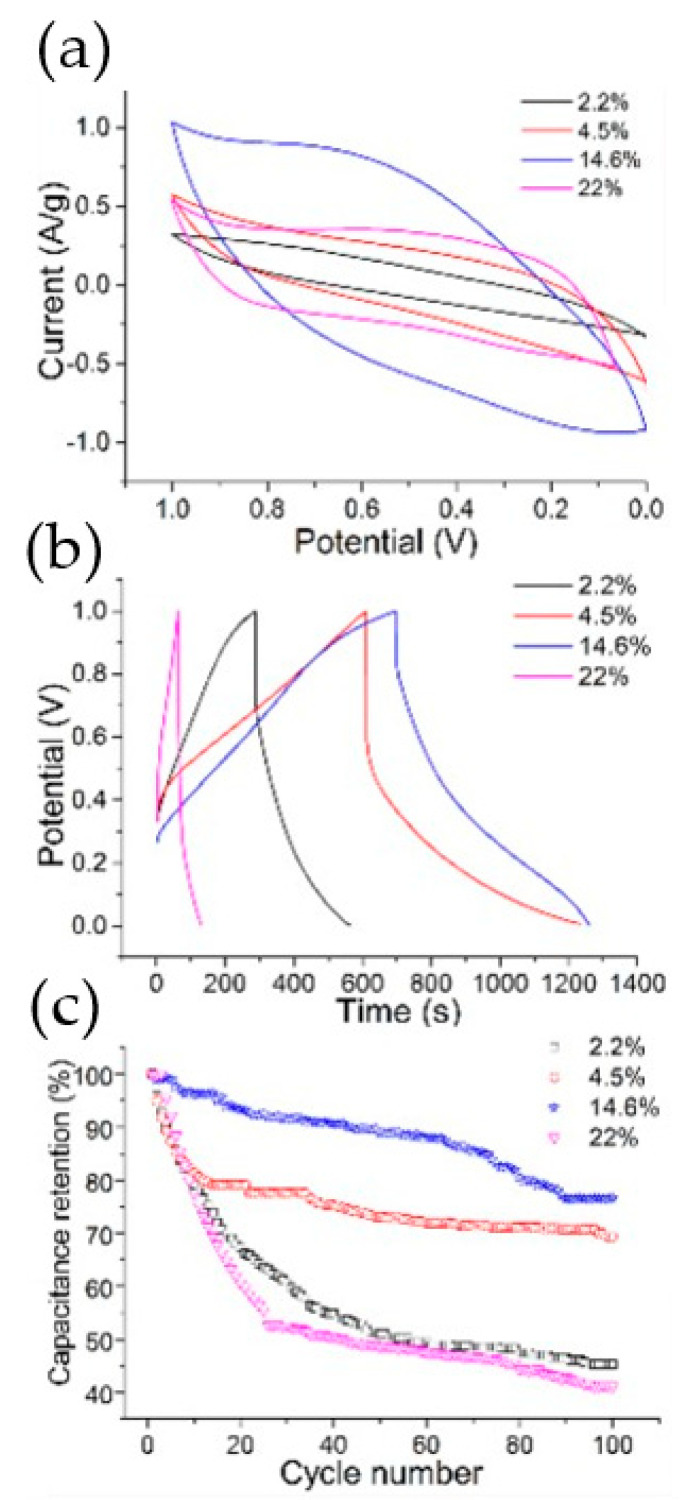
Electrochemical performances of TiO_2_/graphene/PPy with different TiO_2_ content: (**a**) CV curves; (**b**) galvanostatic charge–discharge curves; (**c**) cycle stability. (Reproduced with permission from ref. [43]. Copyright 2015 ACS Publications).

The incorporation of graphene-based composites provides an innovative way for wearable electronics and energy storage devices. Various techniques have been used for the synthesis of these composites. For example, a hydrothermal approach can be applied to fabricate a textile-base graphene composite as an electrode. First, graphene is transferred onto a polyester fabric, and then the graphene/polyester/MnO_2_ composite is placed in an autoclave at 140 °C. Finally, the product is washed with deionized water and dried in an oven. The composite reveals good electrochemical performance with high mechanical stability [42]. TiO_2_ is also another promising electro-active metal oxide. For instance, it was used to fabricate the material for a supercapacitor electrode. The fabrication process of the TiO_2_/graphene/PPy composite for energy applications is as follows. At different temperatures, titian as a starting precursor, was mixed with chemically modified graphene. After drying, electrodeposition of PPy was carried out. As illustrated in Figure 11, the composite revealed increased capacitance and cycling stability [43].

Multiple graphene-based composites including epoxy/graphene, polystyrene/graphene, polyaniline/graphene, nafion/graphene, poly(3,4-ethyldioxythiophene)/graphene, polyethylene terephthalate/graphene, and polycarbonate/graphene nanocomposites have been fabricated through in situ intercalative polymerization, solution intercalation, as well as melt intercalation [44,45]. In addition, a flexible graphene/MnO_2_ composite for paper electrodes was prepared by three steps, during which the GO/MnO_2_ composite was obtained by dispersion. Composite paper was obtained by vacuum filtration followed by thermal reduction [46]. Beside physical synthesis routes, CVD is also a good approach for the fabrication of materials. Therefore, a graphene composite with porous carbon was fabricated via CVD on a Ni gauze substrate, which had excellent compatibility because of the porosity of the composite [47]. Likewise, the hydrothermal method is commonly used on account of its simplicity. For example, ZnFe_2_O_4_ nanoparticles treated with nitrogen-doped reduced graphene were reported as suitable in energy application, specifically for supercapacitors [48]. To attain maximum charge storage and long cycle durability, another composite of 3D graphene/NiOOH/Ni_3_S_2_ was fabricated in two steps. First, 3D graphene was prepared on the surface of nickel foam by the CVD method. Second, the composite was generated by the hydrothermal method [49].

**Figure 12:**
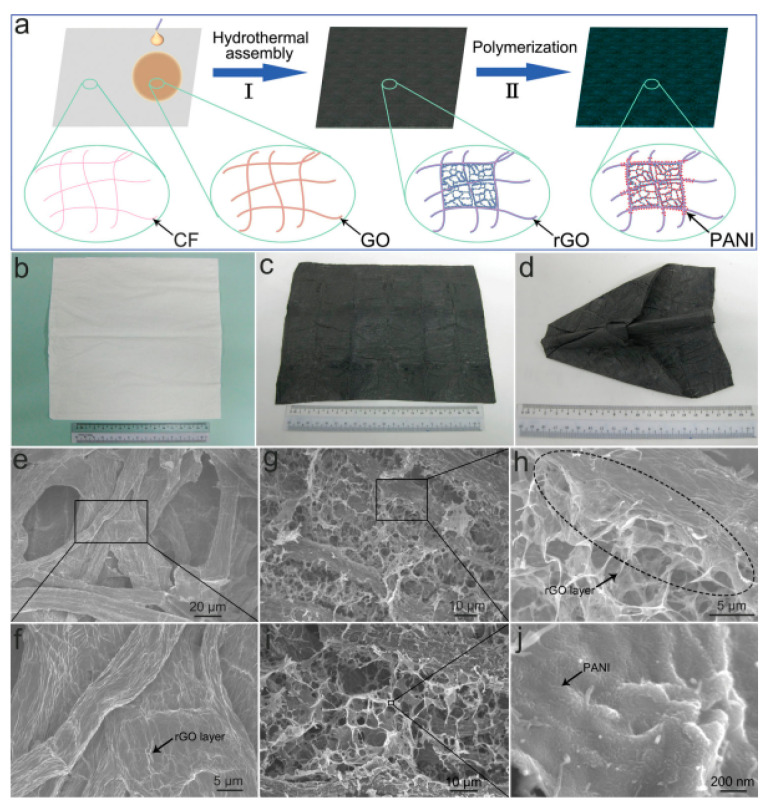
(**a**) Schematic diagram of the preparation of PANI-rGO/cellulose fiber composite paper. Optical images of (**b**) pure cellulose fiber paper and (**c**,**d**) nanostructured rGO/cellulose fiber composite paper. SEM images of (**e**,**f**) rGO-coated cellulose fiber paper, (**g**,**h**) nanostructured rGO/cellulose fiber composite paper, and (**i**,**j**) PANI-rGO/cellulose fiber composite paper. (Reproduced with permission from ref. [51]. Copyright 2014 Wiley Publications).

Although the material choice for certain application remains crucial, the choice of the substrate has a great effect on flexibility. Textile fibers, carbon cloth, and paper pulp have evolved as excellent substrate choices for various graphene-based composites. For example, a graphene-based carbon cloth composite fabricated by the simple brush coating technique showed great properties as an electrode material [50]. Light weight, ultrathin, and flexible electrodes with outstanding mechanical and electrochemical properties are needed of today. As shown in Figure 12, a cellulose fiber-based graphene paper composite was obtained by the dipping and drying method via the hydrothermal route, and possesses environment-friendly and cost-effective features [51].

**Figure 13:**
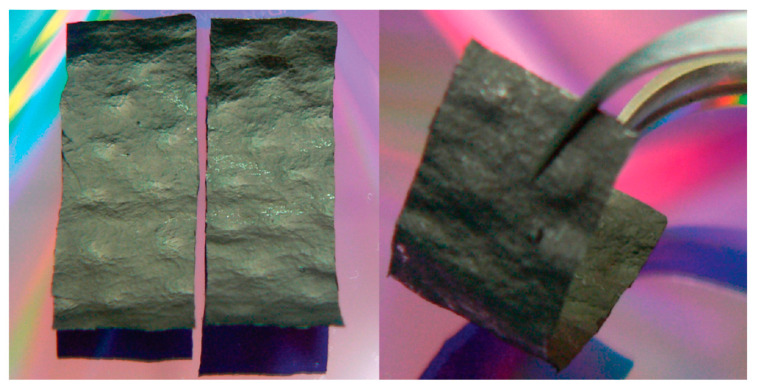
Flexible graphene paper. (Reproduced with permission from ref. [55]. Copyright 2009 ACS Publications.)

The composite of vanadium oxide with graphene paper is binder-free and shows versatility. The fabrication follows an alkaline deoxygenation process, which is more suitable than the chemical reduction of GO to graphene. This flexible composite paper membrane possesses remarkable advantages for double-layered and pseudocapacitive electrodes [52]. In order to avoid toxicity effects, dimethylformamide was utilized instead of hydrazine for the reduction of GO, obtaining outstanding efficiency along with flexibility. Graphene nanosheets were combined with carbon nanofibers to form a composite with enhanced properties via electrospinning, which is favorable for energy applications [53]. In addition to the capacitive properties of carbon-based materials, the mechanical and thermal properties of flexible composite materials obtained from conductive graphene/poly(vinyl chloride) (graphene/PVC) films have also been studied. PVC and graphene sheets were mixed together by liquid dispersion and dripped onto cells, followed by drying in an oven. The acquired composite possesses good thermal stability [54]. Additionally, the properties of conductive polymers like PANI are remarkably enhanced by the addition of graphene-based composites. The formation of a graphene/polyaniline flexible composite can be obtained via in situ anodic electro polymerization. Graphene paper (Figure 13) was directly used as a working electrode in PANI electrolyte, washed, and dried after the complete process, recording a high capacitance [55].

**Figure 14:**
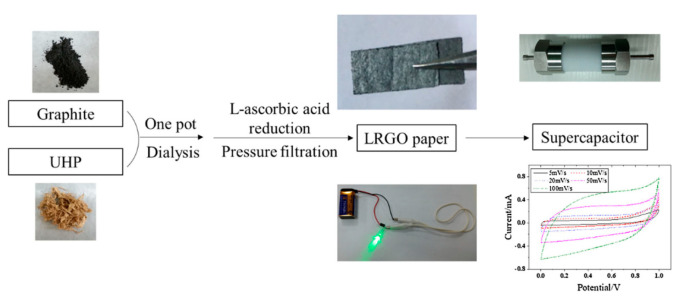
Supercapacitor derived from a conductive paper consisting of a lignocellulose/rGO (LRGO) composite. (Reproduced with permission from ref. [56]. Copyright 2018 Springer Nature Publications).

Moreover, combing graphene with some common substances in nature can lead to composites with outstanding performances. As shown in Figure 14, lignocellulose/graphene conductive paper composite, which worked as a good active electrode, was fabricated through a simple and time-efficient technique by the one-pot method [56].

A nano nickel oxide/graphene PANI composite with enhanced cyclic stability and a high specific capacitance of 92% after 2500 charge–discharge cycles can be applied in the fabrication of energy storage devices [57]. Consequently, graphene-based polymer/metal oxide composite paper electrodes show enhanced electrochemical performance and great potential for application in portable electronics industry.

## 4. Flexible Composites and Applications of GO/rGO

**Figure 15:**
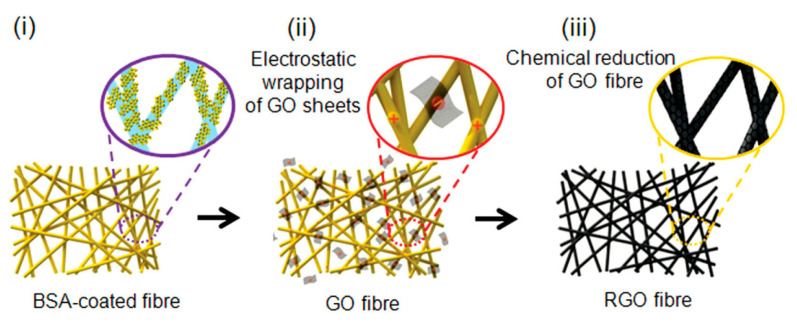
Illustration of the three steps used to prepare rGO/nano yarns (rGO/NYs). (Reproduced with permission from ref. [63]. Copyright 2013 Wiley Publications).

The designed flexible composites should have not only excellent flexibility but also a certain strength which would enable the composites to withstand external environmental factors. Recently, a layered composite of GO/PVC with large mechanical strength fabricated by the vacuum filtration method was reported [58]. These flexible composites composed of GO and rGO are useful in multiple applications including energy storage, water purification, textiles, and robotics. Polymerization was used to prepare PANI nanowires on a GO sheet composite. The obtained products showed excellent performance when used as supercapacitor electrodes [59,60]. Water purification has become another issue in the past few decades. Ongoing research has tackled this problem. For example, a GO-based TiO_2_ composite membrane could be used as a filtration membrane for the removal of water impurities. The composite was fabricated by vacuum filtration and allowed a moderate water purification [61]. Although chemically modified graphene or rGO itself is not very appealing in terms of its properties, these properties can be enhanced in forming composite materials with conductive polymers. For instance, an rGO/polypyrrole nanowires composite fabricated in situ showed better performances than rGO and can be used in the fabrication of portable electronic devices [62]. Yarns were used to produce an electronic textile fabric by coating rGO through electrostatic self-assembly in the presence of adhesive bovine serum albumin. The preparation of the material is shown in Figure 15 [63].

**Figure 16:**
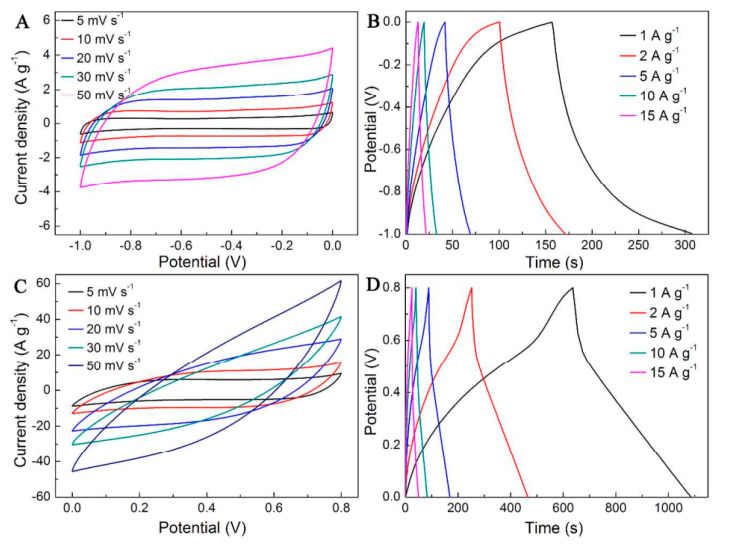
CVs of (**A**) rGO@actived carbon cloth (rGO@ACC) and (**C**) V_2_O_5_/polyindole@ACC. Galvanostatic charge–discharge curves of (**B**) rGO@ACC and (**D**) V_2_O_5_/polyindole@ACC. (Reproduced with permission from ref. [65]. Copyright 2016 ACS Publications).

E-textile has revolutionized the whole flexible and portable device industry with the introduction of additional properties. In addition, a pressed composite of rGO–MnFe_2_O_4_ and polyvinylidene fluoride fabricated by a simple sonication method turned out to be a good absorber of harmful microwaves in the electromagnetic spectrum [64]. Within energy applications, flexible composites of V_2_O_5_/polyindole and activated carbon cloth were used as cathodic and anodic electrodes of an asymmetric supercapacitor. V_2_O_5_ nanostructures were constructed on a carbon cloth by in situ polymerization and showed good cyclic stability on testing (Figure 16) [65].

Paper electrodes for energy application are receiving great attention. A GO solution was prepared by Hummer’s method. Then, GO-based paper electrodes which can act as flexible substrates, actuators, supercapacitor electrodes etc., were fabricated through the steps of vacuum infiltration, spin coating, and drop casting.

**Figure 17:**
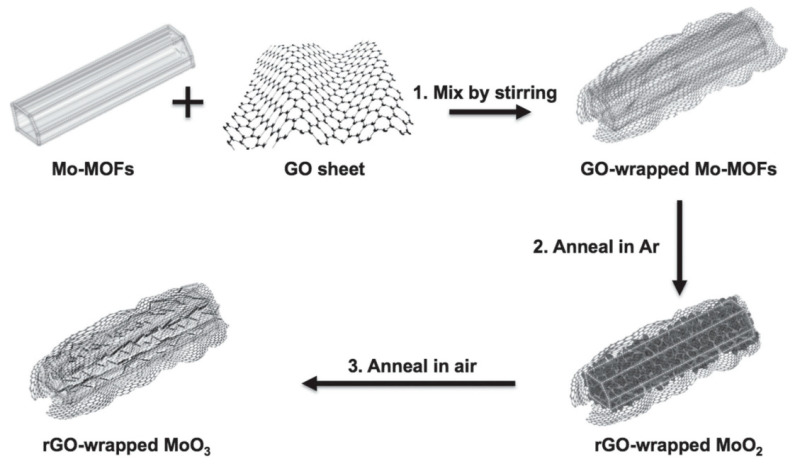
Preparation process of rGO/MoO_3_ composites. (Reproduced with permission from ref. [69]. Copyright 2015 Wiley Publications).

A composite of nickel cobalt oxide/GO was tested as a supercapacitor electrode and revealed a large capacitance of 1211 Fg^-1^. Its fabrication was achieved by coprecipitation using sodium dodecyl sulfate as the template and ammonia as the precipitant [66]. rGO obtained from GO by the hydrothermal route and titanium carbide obtained by selective etching of aluminum were combined by ultra-sonication and filtration, yielding the rGO/titanium carbide composite. CV, GCD, and EIS analysis proved it to be outstanding for electrochemical performance in supercapacitors [67]. Additionally, other carbon-based materials like CNT are extraordinary products due to several characteristics when hybridized with conducting polymers such as MnO_x_ and rGO composites developed by spray coating and electrodeposition. They show a high capacitive behavior and improve the cyclic stability for supercapacitors [68]. Notably, graphene and its derivatives are analogous to other carbon-based materials, providing new perspectives to research. The fabrication techniques are also being modified. Recently, the metal-organic framework template-assisted method was utilized on a large scale and showed great potential for energy applications. In this regard, as shown in Figure 17, rGO/MoO_3_ was reported to be an excellent composite for energy storage in supercapacitors as an electrode [69].

Organic substrates have been widely used in recent studies, but metallic substrates remain an important choice. Copper metallic foil acted as a substrate for the growth of rGO/Cu_2_O through the hydrothermal technique and showed moderate advantages as an electrode in supercapacitors [70]. However, the sol–gel approach is also simple and has been used for the fabrication of rGO-based composites. rGO paper was obtained by a modified Hummer’s method followed by evaporation drying. ZnO was deposited in the form of layers on rGO paper by using a stabilizer through a synthesis process. The composite ZnO/rGO/ZnO has been utilized for supercapacitor electrodes [71]. The choice of the material for positive or negative electrodes plays a vital role in energy devices. A compatible negative electrode material in a supercapacitor for Fe_2_O_3_ nanoparticle clusters/rGO paper was investigated. The composite was synthesized through the hydrothermal technique [72]. Chong et al. [73] prepared an MnO_2_/rGO nanocomposite by a facial one-step electrochemical method. MnO_2_ nanoparticles were uniformly distributed on rGO nanosheets and acted as spacers to prevent rGO nanosheets from restacking. This unique structure provided MnO_2_/rGO with high specific capacitance. Furthermore, the MnO_2_/rGO composite also showed high conductivity and excellent potential cycling stability, and has potential as electrode material for highly stable supercapacitors. In addition to MnO_2_, tungsten oxide (WO_x_) is widely studied as electrode material for supercapacitors. Recently, a W_18_O_49_ nanowires (NWs)/rGO nanocomposite, which can act as the negative electrode in asymmetric supercapacitor devices, was prepared from the precursors WCl_6_ and GO by the solvothermal method [74]. The asymmetric supercapacitor W_18_O_49_ NWs-rGO//rGO showed high specific capacitance and excellent cycling stability. For the fabrication of paper-based electrode, the incorporation of celluloses and pulps is desirable to attain flexibility and stability. GO-based nanocomposite of nanocrystalline cellulose acetate fabricated via stirring and a solvent casting method showed high thermal stability and good mechanical strength [75]. Likewise, cotton pulp was mixed with LiCl in addition with anhydrous DMAc by stirring. Further addition of a GO suspension to cotton pulp resulted in the formation of a cellulose-based composite useful for energy and memory storage [76]. An outstanding anodic electrode material was designed by fabricating a composite of GO and TiO_2_, whereas further reduction of the composite to rGO/TiO_2_ was obtained by stirring and drying. Anatase TiO_2_ exhibits higher power and energy density than other conventional metal oxides [77]. In comparison with cellulose, the residual paper pulp is more stable. As a consequence, it can be used in the fabrication of rGO-based flexible composites. First, the paper pulp was stirred in stable solvent and then it was mixed with GO. Next, the suspension was infiltrated with and reduced by hydrazine vapors at a certain temperature via the drop casting technique. The obtained composite possessed better performance compared with cellulous-based composites when applied in flexible electrodes [78]. Altogether, natural fiber-based GO/rGO paper composites have been proven to have excellent performance in multiple applications, especially in energy storage and conversion devices in the modern portable device industry.

## 5. Conclusions and Perspective

With the fast development of portable, wearable, and lightweight electronic devices, highly efficient and flexible energy strategies are urgently needed. In order to achieve this goal, it is crucial to explore novel materials. Graphene has attracted tremendous attention in the field of material science due to its outstanding properties since it was first exfoliated in 2004. This review aimed to outline the different fabrication methods and applications of graphene-based materials, especially for flexible, portable, environment-friendly, and cost-effective energy storage and conversion devices. Some representative methods used to prepare the composites based on graphene and the corresponding applications are listed in Table 1. The outstanding performances of these composites are due to the special structure and excellent properties of graphene, as well as the ease of functionalization of GO and rGO. Graphene-based materials show great application potential in flexible devices. In addition, they further promote the miniaturization and portability of devices and have a huge effect on human life. For example, integrating graphene-based energy storage into wearable devices is promising for human health monitoring. Graphene-based composite membranes also show potential applications in water purification and can be used to remove dyes molecules in water. The progress in flexible composites based on graphene and its derivatives is rapid, and some achievements have been made in recent years. However, to realize graphene‘s practical applications, there are still many challenges to solve. For example, the large-scale production of graphene with high quality and uniform structure is still a big challenge. There are many techniques that can be used to prepare graphene, such as exfoliation from graphite and CVD techniques, but they are cumbersome, time-consuming, and expensive. Furthermore, the complicate transfer process further limits the wide application of graphene. Chemical oxidation of graphite is the most widely used method way to prepare graphene derivatives. However, the synthesis and purification procedures of the oxidation of graphite are complex and risky. It is hard to precisely control the compositions and sizes of graphene sheets, which heavily affects the performance of the composites. In addition, in order to synthesize composites with excellent performances, interfacial interactions between graphene or its derivatives and other functional materials need to be systematically studied. Although difficulties and challenges still exist, with the development of science and technology, more and more technically feasible strategies will be explored. We believe that flexible graphene-based devices and systems will emerge as essential instruments in our daily lives.

## Figures and Tables

**Table 1:** Preparation and application of graphene-based composites.

Carbon-Based Material	Composites	Preparation Methods	Applications
graphene	poly ethylenedioxythiophene-graphene	electrochemically deposition	energy storage devices [32]
	(PEDOT:PSS)/graphene	in situ polymerization	energy harvesting systems [35]
	graphene/MnO_2_/CNTs	chemical co-precipitation method	energy storage devices as a robust electrode [38]
	graphene/PANI	electropolymerization	paper electrode [39]
	graphene/MnO_2_	electrodeposition	asymmetric supercapacitor [41]
	TiO_2_/graphene/PPy	electrodeposition	supercapacitor [43].
GO/rGO	PANI/GO	polymerization	supercapacitor electrodes [59,60]
	GO based TiO_2_ composite membrane	vacuum filtration	water purification system [61]
	nickel cobalt oxide/GO	coprecipitation	supercaps electrode [66]
	rGO/polypyrrole nanowires composite	in situ route	portable electronic devices [62]
	rGO/Cu_2_O	hydrothermal technique	supercaps [70]
	paper pulp/rGO	drop casting technique	flexible electrode [78]

## Data Availability

No new data were created or analyzed in this study. Data sharing is not applicable to this article.
